# Assessing Depression in Cardiac Patients: What Measures Should Be Considered?

**DOI:** 10.1155/2014/148256

**Published:** 2014-02-06

**Authors:** M. Ceccarini, G. M. Manzoni, G. Castelnuovo

**Affiliations:** ^1^Psychology Department, University of Bergamo, 24129 Bergamo, Italy; ^2^Istituto Auxologico Italiano IRCCS, Ospedale San Giuseppe, 28922 Verbania, Italy; ^3^Psychology Department, Catholic University of Milan, 20123 Milan, Italy

## Abstract

It is highly recommended to promptly assess depression in heart disease patients as it represents a crucial risk factor which may result in premature deaths following acute cardiac events and a more severe psychopathology, even in cases of subsequent nonfatal cardiac events. Patients and professionals often underestimate or misjudge depressive symptomatology as cardiac symptoms; hence, quick, reliable, and early mood changes assessments are warranted. Failing to detect depressive signals may have detrimental effects on these patients' wellbeing and full recovery. Choosing gold-standard depression investigations in cardiac patients that fit a hospitalised cardiac setting well is fundamental. This paper will examine eight well established tools following Italian and international guidelines on mood disorders diagnosis in cardiac patients: the Hospital Anxiety and Depression Scale (HADS), the Cognitive Behavioural Assessment Hospital Form (CBA-H), the Beck Depression Inventory (BDI), the two and nine-item Patient Health Questionnaire (PHQ-2, PHQ-9), the Depression Interview and Structured Hamilton (DISH), the Hamilton Rating Scale for Depression (HAM-D/HRSD), and the Composite International Diagnostic Interview (CIDI). Though their strengths and weaknesses may appear to be homogeneous, the BDI-II and the PHQ are more efficient towards an early depression assessment within cardiac hospitalised patients.

## 1. **Introduction**

A significant number of patients with heart disease suffer from depression at some point during the course of their illness [1, 2]. According to the most reliable estimate, about 15–20% of hospitalised cardiac patients meet diagnostic criteria for a major depressive disorder and an even higher percentage (from 25% to 65%) reported at least one depressive symptom [3, 4]. In comparison, the annual prevalence of major depression in the general adult population is around 5%, while it rises up to 10% if we consider the whole lifespan [5].

More or less severe depression is mainly found in hospitalised patients who had myocardial infarction [6], though it is frequently observed in patients with unstable angina [7] or heart failure [8, 9], amongst those who had a coronary artery bypass intervention [10] and in patients who had cardiovalvular surgery [11]. However, according to other authors, depression is more frequently found following cardiac surgery rather than after a heart attack. In addition, patients who develop a depressive disorder after a cardio-surgical intervention remain depressed for longer and only improve if they receive antidepressants, while depression following a myocardial heart attack tends to heal spontaneously [5].

Longitudinal studies have generally demonstrated that depression can last many months after the acute phase of a heart disease and that it causes significant loss in functioning beyond what is expected after the illness itself. In some cases, depression can evolve into disability [12], it can originate a new acute cardiac event and, apart from a few exceptions [13], it seems to increase the risk of premature death during the first year after the acute event, for both minor and major depression [6, 14, 15]. It can be inferred from collective data that cardiac patients with major depression have an increased risk of three to four times higher to prematurely die after an acute cardiac event than patients who do not suffer from depression [16].

Depression seems to be a cardiovascular risk factor in healthy subjects as well [16] as demonstrated by a meta-analysis conducted in cardiologically asymptomatic individuals. The eleven prospective studies found that the presence of a depressive disorder was associated with major cardiac events with a relative risk of 2.69 [17], compared to the Framingham Heart Study, in which hypertension was associated with the same major cardiac events with a relative risk of 1.92 [18]. In a normative aging study, 735 men over sixty with no coronary/artery diseases were evaluated to verify the presence of anxiety symptoms; after 12 years, the anxiety level measured at baseline proved to be associated with myocardial infarction with a relative risk of 1.43 [19].

Hence, the health status of patients during the weeks and months following the acute cardiac event is often the result of a cardiovascular pathology process already developing from some time before. In fact, patients are often confronted with the risk of dying, undergoing other acute critical events, and/or being impaired for life [1]. They soon discover that daily activities which did not represent a problem before the disease (physical-motor activities in particular) become difficult, impossible to perform, or even prohibited because of the cardiovascular risk involved. Other activities such as working and/or domestic/familial skills can be severely compromised with serious repercussions on the personal and social identity [1]. Overall, a real crisis begins and develops affecting the mind, the body, and the person as a whole. The individual's psychological organization in a wide range of cognitive, emotional, and behavioral symptoms is highly perturbed. In most patients, such symptoms are resolved fairly quickly. The adjustment process depends on the necessary time for each individual to complete physiological and bio-psycho-social adaptation with respect to the disease and its consequent lifestyle changes. The latter represent a normal reaction to stressful events [15].

However, in a fair percentage of patients, these symptoms can last longer, get worse, or later become an overt psychopathological syndrome (i.e., adjustment disorders, mood disorders, and posttraumatic stress diseases) and this depends on their personal resources, psychological characteristics, sociocultural environment, and their disease peculiarities. A cognitive adaptation theory formulated by Taylor and Brown [20] explains that the majority of individuals are convinced to have control over stressful events and over reality; they nurture positive expectations about the future and have a positive self-image. Such cognitions, though often illusory, are adaptive and functional with regard to mental health. A critical event, like a stroke, an acute coronary attack, or a serious disease in general, can hardly strike upon these positive assumptions about the greater world and the self to the point of completely destroying them and throwing the person into a deep state of insecurity and uncertainty about the future [21, 22].

Mild and severe feelings of depression, anxiety, and anger, along with their related cognitive correlates, painfully burst into the individual's experience and accompany him/her throughout the adaptation path. In this process, several variables come into play: from personal characteristics to the disease severity, from personal coping styles to distal or closer social contexts, and from individual cognitive schemata and reality construction to the quality of the social support received. That is to say that an optimistic, self-confident individual with a high self-efficacy and self-control, a good coping capacity, and a supportive and empathic social environment is more likely to quickly and positively adapt to the disease. A pessimistic, insecure, helpless, and discouraged individual with little or inadequate coping skills, who is socially isolated or with little or no social support at all, is unlikely to adapt to the disease. Hence, he/she runs the risk of developing an adaptation disorder first and a possible mood disorder later. The cardiac patients' main task is therefore rebuilding or accommodating to the functional cognitive assumptions which have been undermined, restoring the perception of control over the situation [23]. At times, the adaptation process may be blocked or lead towards the development of dysfunctional cognitive schemas which Beck's cognitive theory places at the base of depression and anxiety, as opposed to developing positive functional assumptions associated with wellbeing.

Despite the prognostic importance of depression in cardiac patients, an estimate suggests that depressive symptoms and disorders are diagnosed and treated in less than 15% of cases [24]. Ziegelstein et al. [25] evaluated the ability of healthcare staff to recognize the presence or absence of depressive symptoms in hospitalised patients following a myocardial infarction. They discovered that with no specific screening tools, results reached up to 75% of false negatives. Recognising depressive symptoms in cardiac patients is even more difficult. This is due to the fact that they are unaware of being depressed as they attribute to their heart disease classic depression symptoms mistakenly judging them as cardiac ones. There are several signs that should prompt the suspicion of witnessing a depressed patient, beyond his/her assertions. These are chronic fatigue, irritability, being prone to anger, disturbed sleep, social withdrawal, lack of compliance with medical and behavioural requirements, unjustified medical checks, little or no progress during rehabilitation, and so on [7].

Interestingly, during and after an acute cardiac event, male patients often feel angry. Due to cultural and social reasons, anger, especially in males, works as a reaction to depressive covert feelings that are not accepted. Thus, when patients are angry, both health practitioners and family tend to minimise and underestimate such responses rather than understanding if the emotion experienced is a sign of depression [5]. In order to overcome these diagnostic obstacles or at least to avoid excessive assessment errors, it is important to rely on questionnaires, structured interviews, and checklists that have previously demonstrated good levels of validity and reliability with reference to the specific population, that is, the heart disease population. Many clinician-rated and patient-rated instruments have been developed to measure depression in clinical trials in the last twenty years; however, depression screening in cardiovascular patients does not always correspond to an early, accurate use of suitable tools.

In this paper, the analysis of eight major assessment instruments the Hospital Anxiety and Depression Scale (HADS), the Cognitive Behavioural Assessment Hospital Form (CBA-H), the Beck Depression Inventory (BDI), the two and nine-item Patient Health Questionnaire (PHQ-2, PHQ-9), the Depression Interview and Structured Hamilton (DISH), the Hamilton Rating Scale for Depression (HAM-D/HRSD), and the Composite International Diagnostic Interview (CIDI) will be performed in order to provide a comparison between them and to identify which one is more suitable in detecting mood changes within the cardiac patients hospitalised population. The aim of this work is to go through the fundamental steps that have most commonly been used to evaluate depression in cardiac hospitalised patients. The selection of the eight tools mentioned above refers to what has been suggested by international studies on heart disease patients at the National (Italian), European, and American level. The present study follows the recommendations of guidelines regarding the best path leading towards high screening quality.

Thus, the authors' search strategy strictly refers to the indications specified by the Italian National System of guidelines (SNLG), the Italian Institute of Health (2005), the American health institutes (NHI), the National Heart, Lung and Blood Institute (2006), and finally the European guidelines for the prevention of cardiovascular disease in the clinical practice published by the European Cardiology Society (2007). The questionnaires included in this work refer to those expressively suggested in previously mentioned sources. Moreover, in this paper the best instruments amongst the eight listed in the four major guidelines above which are more likely to overcome the danger of underestimating a depressive condition in heart disease patients are outlined.

The following is therefore an overview of the tools taken into consideration by Italian, American, and European recommendations on cardiac patients depression screening. The authors' aim is to highlight which of the eight instruments is mostly appropriate, rapidly administered, short, simple, and useful in identifying psychological aspects related to depressive symptoms underlying the condition of hospitalised cardiac patients rather than concentrating on general areas of distress or misjudging mood disorders for other medical conditions. Thus, strengths and weakness of the questionnaires, semi-structured or structured clinical interviews analysed in this review will be pointed out. Hence, the most recommendable tools will be clearly identified. It is important to verify which instrument proves itself to be more useful to evaluate depression since mood disorder screening is fundamental in later providing patients with the best possible psychological support and most suitable treatment.

## 2. The Assessment of Depression in Cardiac Patients

In the international literature the simplest and most widely used tool is the Hospital Anxiety and Depression Scale (HADS) [26]. The questionnaire was designed to provide a reliable tool within the clinical practice and it is composed of a fourteen item scale of which half identify the level of anxiety and the other half relate to depression. The authors created this outcome measure specifically to avoid excessive reliance on other aspects which are intertwined with both anxiety and depression but are yet different (i.e., common somatic symptoms of illness, fatigue, insomnia, or hypersomnia). The aim this psychometric tool was to detect of anxiety and depression in individuals with relevant physical health problems [26].

More specifically, items of the Hospital Anxiety and Depression Scale (HADS) are scored from 0 to 3 on a Likert scale with a final score ranging from 0 to 21 for either anxiety or depression. There are a large number of studies that have explored the underlying factor structure of the Hospital Anxiety and Depression Scale (HADS), many of which support the two-factor structure, although others suggest a three- or four-factor structure, while some argue that the tool is best used as a unidimensional measure of psychological distress [27]. The Hospital Anxiety and Depression Scale (HADS) is a questionnaire that performs well in screening for separate dimensions of anxiety and depression in cases of anxiety disorders and depression in patients from nonpsychiatric hospital clinics and it seems to have at least as good screening properties as similar, but more comprehensive instruments used for identification of anxiety disorders and depression [28].

The utility of the Hospital Anxiety and Depression Scale (HADS) as a screening instrument for coronary care patients following acute myocardial infarction (MI) has been investigated by Martin et al. [29]. Results demonstrated that the questionnaire has sound psychometric properties in MI patients over three different time frames (after 1 week, 6 weeks, and 6 months) through a confirmatory factor analysis. Internal and test-retest reliabilities of both total and subscale scores were generally good as the questionnaire allowed to determine subscales factors assessing dimensions of anhedonia, psychic anxiety, and psychomotor agitation. The Hospital Anxiety and Depression Scale (HADS) is hence a reliable instrument useful to screen and evaluate post-MI patients for symptoms of psychological distress [29].

Nonetheless, patients reliably and validly reporting on a continua of anxiety and depressive symptoms appear to be rather arbitrary due to the constriction of breadth of content, which interferes with providing an efficient first stage screening to determine whether they meet formal diagnostic criteria for an anxiety or depressive disorders. That is to say that the Hospital Anxiety and Depression Scale (HADS) has an idiosyncratic conception of the core symptom of depression as being anhedonia, leading towards oversampling and less applicability to the mild to moderate range of sad or blue depression symptoms. The tool may therefore be weak in detecting mood disorders in contexts where many medically ill patients without psychopathological issues can be found, including cardiac units. Such matter limits a refined discrimination of symptoms severity [30].

In 2005, the national guidelines on cardiac rehabilitation and secondary prevention of cardiovascular diseases were published in the Italian National System of guidelines (SNLG) of the the Italian Institute of Health with an entire chapter dedicated to psychological and educational interventions. In the document it is stated that “an agreement regarding the instrument more appropriate to use for the measurement of “psychological wellbeing” was not reached yet.” Further suggestion coming from the Italian Institute of Health guidelines on the assessment of depression in cardiac patients are proposed in the guidelines appendix, stating that the Cognitive Behavioral Assessment Hospital Form (CBA-H), which is the most commonly used instrument to assess depression which is very similar to the Hospital Anxiety and Depression Scale (HADS) and the Beck Depression Inventory (BDI) [31] should be strongly considered. In addition, the document specifies that for depression as well as for anxiety, screening should take place at the beginning of the rehabilitation and also 6–12 weeks after the acute event in order to identify patients persisting with those symptoms [32].

The Cognitive Behavioral Assessment Hospital Form (CBA-H) was developed by Bertolotti and colleagues [33], to allow a quicker assessment within the hospital or health context. The questionnaire has 147 items structured with a true/false answering system which is rather simple, and it takes about 10–20 minutes to be completed. The CBA-H is composed by four cards: A, B, C, and D which take into account different time lags, hence, discriminating between emotional states and behavioural changes related to the recent hospitalization or health diagnosis and the patient's preexisting characteristics.

Card A contains 21 items focusing on the present time (i.e., hospitalization or diagnosis communication), evaluating anxiety and depression states and fears and worries. Card B contains 23 items asking about the previous three months investigating on dysphoria and on other psychophysiological disorders and stress. Card C contains 61 items focusing on the period of time prior to the disease and it asks a self-reported patient description of his/her stable character and behaviour such as introversion/extroversion, neuroticism, social anxiety, speed and impatience, job involvement, hostility, hard driving, and irritability. Card D contains 47 items on biographical information about general lifestyle (work, affective and sexual life, smoking, eating and drinking, sleep quality, and physical exercise) and health risk factors (stressful events). The entire questionnaire scoring includes both quantitative measures and in depth examination patterns as well as suggestions for further interventions within the health psychology and behavioural medicine fields. The tool includes a software program which creates a global report on the patient's psychological profile and hypothesis for the additional clinical interview.

Although the Cognitive Behavioral Assessment Hospital Form (CBA-H) can be considered a valid and complete tool for general psychological distress screening within the hospital context, it must be viewed as a battery of different tests which do not specifically address mood disorders and depressive symptomatology. In fact, only Card A is specifically structured to analyse the patients' situational psychological state, such as those emotional reactions that the hospitalised individual experiences at the time of completion of the tests. This part of the tool is particularly suitable for patients who accesses a rehabilitation cardiac program as it enquires about feeling sheltered and about the experiences regarding the illness. However, it may not be enough for clinicians to use the entire Cognitive Behavioural Assessment Hospital Form (CBA-H) or to fully rely on it when assessing a target condition possibly accompanying cardiac patents, such as depression.

When it comes to the Beck Depression Inventory (BDI), it is a much more renowned gold-standard scale [31], designed to measure depressive symptoms severity at the present time. The original self-rating Beck Depression Inventory (BDI) includes 21 items concerning different symptom domains, with four possible answers describing symptoms of increasing severity associated with a score ranging from 0 to 3. The questionnaire was later reviewed into the Beck Depression Inventory-IA form [34] and then a second version was made, the Beck Depression Inventory-second edition (BDI-II) with an extended rating from 1 to 2 weeks. The more recent version of the Beck Depression Inventory (BDI) was created following the publication of the DSM-IV [35]. It includes four new items added to better pertain to the manual depression criteria, namely, agitation, worthlessness, concentration difficulty, and loss of energy. Some Beck Depression Inventory-IA form items such as weight loss, body image change, work difficulty, and somatic preoccupation were eliminated as they were not so related to the overall severity of depression.

The Beck Depression Inventory has been extensively studied. Results have been consistently positive and the Beck Depression Inventory is now known to correspond with over 90% of clinical diagnoses for patients suffering from depression. It is also widely agreed that the test adequately covering the range of conditions commonly exhibited by those with depression, accurately measuring the severity of the ailment, while meeting with recent medical and psychological standards [36]. Some may argue that because the Beck Depression Inventory is self-reported, there is a possibility that participants may exaggerate giving their answers. This could be applicable especially in hospitalised heart disease patients who may feel more despondent than they would normally feel. Nonetheless, it is important to point out that the Beck Depression Inventory II (BDI-II) can only be used to measure the severity of depression and not strictly as a diagnostic tool as such. Moreover, it is particularly useful in conjunction with other tests in order to provide a proper analysis of patients' current mental state. It measures depression intensity on a weekly bases, transversely to the types of depression and different diagnostic categories, as the depressive condition is considered as a psychological trait, therefore nonpathological. That is to say that the score can be analysed in a cognitive-affective subscale and a symptomatic-somatic one. Also, the Beck Depression Inventory II (BDI-II) indications of a clinical cut-off alarm are very clear.

In 2006, one of the American health institutes (NHI), the National Heart, Lung and Blood Institute published a document with some recommendations for the evaluation and treatment of depression in cardiac patients defined by an interdisciplinary team especially created for the matter. The paper recommends the use of the Beck Depression Inventory for epidemiological studies, the Patient Health Questionnaire (the two-items form) for initial screening, and the structured interview formulated by the ENRICH Study group [37], namely, the Depression Interview and Structured Hamilton (DISH) for the diagnostic assessment and the Hamilton Rating Scale to evaluate change and symptomatic remission [38].

In the following year, in 2007, the fourth updated edition of the European guidelines for the prevention of cardiovascular disease in the clinical practice was published by the European Cardiology Society and nine other institutions incorporated in a single task force. In the final document, along with the classic risk factors such as hypertension, diabetes, and obesity, psychosocial factors were also considered. The assessment of depression using simple and straightforward instruments was also suggested [39].

An example of a direct and easy-to-use tool is represented by the two-items form of the Patient Health Questionnaire, the PHQ-2, a yes/no screening tool enquiring about the patient's past 2 weeks and asking if she/he has noticed little interest or pleasure in doing things and/or has felt down, depressed, or hopeless. If the answer is “yes” to either question, professionals qualified in the diagnosis and management of depression should refer for more comprehensive clinical evaluation using the nine-item version of the questionnaire, the Patient Health Questionnaire Nine (PHQ-9), [40]. The latter investigates the same dimensions of the two-item version, though including questions on the sleep overall quality, energy level, appetite, feeling bad about the self, concentration problems, communication or movement speed rate, and the eventual presence of self-harming or self-negative intentions. Questions are scored 0 for not at all, 1 for several days, 2 for more than half the days, and 3 for nearly every day. Adding together the item scores it is possible to obtain a total score which represents the level of depression severity [41–44].

Most patients are able to complete the Patient Health Questionnaires in more or less than five minutes with no assistance, yielding a provisional depression diagnosis and a severity score which can be used for treatment selection and monitoring. The Patient Health Questionnaire Nine (PHQ-9) has been shown to have reasonable sensitivity and specificity for patients with coronary artery disease. Nonetheless, for those who display mild symptoms, it would be better to recall for a subsequent visit or followup, while for those with high depression scores, a specialised practitioner should review the answers with the patient to gain a clearer picture. On the whole, the two-items form of the Patient Health Questionnaire (PHQ-2) also shows good specificity but it has poor sensitivity in patients with coronary artery disease. Similar results have also been found for the nine-item version of the Patient Health Questionnaire (PHQ-9), [42]. The latter seems to be a useful tool to recognize not only major depression but also subthreshold depressive disorder in the general population [29]. Moreover, Patient Health Questionnaire Nine (PHQ-9) is half the length of many other depression measures and consists of the actual nine criteria on which the DSM-IV diagnosis for mood disorders is based.

Particularly, the Patient Health Questionnaire Nine (PHQ-9) has a double objective: establishing a provisional depressive disorder diagnosis and symptom severity rating in order to carry on treatment, since a 5-point score or above falls into the questionnaire global score and qualifies as a clinically significant response to depression intervention. In fact, each 5-point change on the Patient Health Questionnaire Nine (PHQ-9) represents a moderate effect size on multiple domains of health-related quality of life and functional status. A score of less than 10 qualifies as a partial response, while a score of less than 5 counts as remission. It is important to keep in mind that such values must be viewed in a rules of thumb logic, hence, requiring clinical evaluation of the individual heart disease patient. Brevity coupled with its construct and criterion validity makes the Patient Health Questionnaire Nine (PHQ-9) an attractive, dual-purpose instrument for making diagnoses and assessing severity of depressive disorders, particularly in the busy setting of clinical practice [41].

Further indications on assessing depression in cardiac patients come from the renowned Enhancing Recovery in Coronary Heart Disease Patients (ENRICHD) trial which examined the effects of cognitive behavioural therapy plus adjunctive sertraline treatment in case of insufficient response on depression, and cardiac outcomes in postmyocardial infarction (MI) patients. The research represents a target study as it demonstrated that a Cognitive Behavioural Therapy treatment provided to a very large sample of over two thousands cardiac, depressed patients did help participants reduce general depressive symptoms but failed to determine a significant reduction in death rates in the months following the cardiac episode, compared to patients who received a traditional psychological treatment [45]. For the ENRICHD study a semistructured interview was specifically formulated, the Depression Interview and Structured Hamilton (DISH) designed to minimize respondent burden without losing thoroughness nor accuracy of the information was gathered [37].

The Depression Interview and Structured Hamilton (DISH) is suitable to screen cardiac patients for depressive disorders, diagnose major and minor depression or dysthymia according to the DSM-IV criteria, rate the severity of depression on a structured version of the Hamilton Rating Scale for Depression (HRSD), and gather the history and development of depression. The interview is divided into three sections. The first is the “Optional Opening Questions” and it is comprised of open-ended questions in order to develop therapeutic alliance and encourage the patient to open up. The second section is on the 17-item Hamilton scale and it is called the “Current Depression Symptoms.” This part of the interview includes criteria needed to diagnose major and minor depression or dysthymia and to evaluate depression severity in the past week. The depressive symptomatology is coded absent, subthreshold, present, or present due to direct physiological effects of the cardiac condition or treatment. Symptom vary according to how long they last in weeks and they are coded separately according to the number of days they have been present for, though less than two weeks [37].

Moreover, in the second section, compulsory questions are verbatim administered with the aim of verifying the existence of a DSM-IV depressive disorder and obtaining an Hamilton Rating Scale for Depression (HRSD) score. Some assess atypical features of depression (increased appetite, weight gain, and hypersomnia) and bereavement, while others are worded according to the patient's personal preference for the symptoms terminology (i.e., “feeling sad/depressed” is referred to as “feeling down” or “blue”), leaving the interviewer to assess whether the patient's terms intertwine with the Diagnostic and Statistical Manual of Mental Disorders (DSM IV) criteria. Optional questions clarify the context, frequency, severity, duration, and qualitative features of depressive symptoms. The first items of the “Current Depression Symptoms” facilitates a rapid screening of nondepressed subjects, directly assessing the main symptoms of depression (dysphoria and loss of interest or pleasure in usual activities). Nonetheless, for cardiac patients who wish to approach their somatic symptoms first, question order may be changed to promote a better therapeutic alliance and self-disclosure. The section ends with a brief assessment of signs or symptoms of major psychiatric disorders (i.e., paranoia, delusions, hallucinations, hypomania, and confusion), aiming at verifying severe psychiatric comorbidity [37].

The third and last part of the Depression Interview and Structured Hamilton (DISH) is the Psychiatric History section in which most items enquire about the patient's previous personal history and on major depression. Along with familial history of depression, the section asks about the number of past episodes, the age at first onset and at first onset of the last episode, history of bipolar disorder, alcoholism, and other disorders that might be worth of clinical attention. A Longitudinal Course Chart is used to document the interim course of any possible depressive disorders from the interview baseline to any eventual exacerbations, remissions, relapses, recurrences, or new depressive episodes [37].

The Hamilton Rating Scale for Depression (HAM-D or HRSD) is one of the most popular and old scales specifically developed to assess depression severity. From its original version, namely, the Hamilton Rating Scale for Depression (HAM-D) the last four items (diurnal variation, depersonalization/derealization, paranoid symptoms, and obsessive compulsive symptoms) were eliminated as they concerned symptoms later considered to be uncommon or not representative of depression severity as such [46]. The 17-item version of the test has become the standard for clinical trials and, over the years, it proved to be the most widely used scale for controlled clinical trials in depression. Nonetheless, some investigators believe that such a reduced version presents some limitations such as noninclusion of all symptom domains of major depressive disorder, reverse neurovegetative symptoms in particular, some items measuring constructs irritability and anxiety, and loss of interest and hopelessness which differ from “pure” depression and different rating attributed to different symptom domains (insomnia coded up to 6 points, while fatigue only up to 2) [47].

Time administration of the interviews is around 12 minutes without taking into account that its duration may be longer due to psychomotor retardation or any other impediment given by depression or overall health conditions. The total score is obtained by summing up the score of each item, ranging from null to four, that is to say, from no symptoms to mild, moderate, or severe depression or from null to two, which corresponds to absent, slight or trivial, and clearly present depression. For the 17-item version, scores can range from 0 to 54.

Moreover, for most clinicians scores between 0 and 6 do not indicate the presence of depression, scores between 7 and 17 indicate mild depression, scores between 18 and 24 indicate moderate depression, and scores over 24 indicate severe depression. A total Hamilton Rating Scale for Depression (HAM-D or HRSD) score of 7 or less after treatment is a classic indicator of remission. A decrease of half or more symptoms from the interview baseline during the course of a depression treatment is considered an indicator of clinical response, or in other words, a clinically significant change [47]. Because of its widespread use over the course of decades, the Hamilton Rating Scale for Depression (HAM-D or HRSD) is the most popular measure to verify depression severity in the history of MDD trials, and it is very familiar to most clinical researchers in the area of depression [38].

A recommendation when it comes to psychosocial screening is to provide for all patients with a heart disease the use of clinical interviews and standardized questionnaires, for which Albus et al.'s publication [48] is worth mentioning. In the document three methods to evaluate psychosocial factors in cardiac patients are presented: first, the standardized and structured interview, secondly, the self-administered questionnaires, and thirdly the clinical interviews, even those just composed by a single question. Structured interviews are obviously the gold-standard to diagnose psychopathological disorders but they require a high administration time which could represent a limitation in the clinical practice. Albus and colleagues [48] suggest to use the Composite International Diagnostic Interview (CIDI) a structured comprehensive interview which closely relates to the syndromic definitions of different mental disorders proposed by the tenth edition of the International Classification of Diseases (ICD-10), and in the fourth edition of the Diagnostic and Statistical Manual of Mental Disorders (DSM IV).

The Composite International Diagnostic Interview (CIDI) is a comprehensive and fully standardised diagnostic instrument containing 276 symptom questions, many of which are used to evaluate symptom severity, help-seeking behaviour, psychosocial impairments, and single episode-related matters. Although primarily intended for use in epidemiological studies of mental disorders, it is also being extensively used for clinical and other research purposes. The Composite International Diagnostic Interview (CIDI) was judged to be acceptable for most subjects and was found to be appropriate in different kinds of settings and countries [49]. Although it is a soundly structured interview, the Composite International Diagnostic Interview (CIDI) is designed to be used by trained interviewers who are not clinicians and it is therefore advantageous in administration flexibility. It comprises 11 diagnostic sections, which may be administered independently, covering many areas.

The interview is modular and covers somatoform disorders, anxiety disorders, depressive disorders, mania, schizophrenia, eating disorders, cognitive impairment, and substance use disorders. Two questions are used to assess drug and alcohol abuse, a screening module and 40 sections focusing on diagnoses (22 sections), functioning (four sections), treatment (two sections), risk factors (four sections), sociodemographic correlates (seven sections), and methodological factors (two sections), [50]. Although the Composite International Diagnostic Interview (CIDI) may underdiagnose disorders compared to other instruments such as the Structured Clinical Interview for DSM-IV (SCID), it performs well as a research instrument to diagnose major depression in MI patients [51]. Though the Composite International Diagnostic Interview (CIDI) allows us to provide a psychiatric diagnosis through computerized algorithms in accordance with the International Classification of Diseases 10th edition (ICD-10) and the American Psychiatric Association Diagnostic and Statistical Manual of Mental Disorders 4th edition (DSM-IV) some authors criticised the tool as it may force a range of complex experience into a fixed-choice interview format and it may not prove to be a sound instrument within different cultural contexts [52].

All in all, self-administered questionnaires are much more advantageous from a time consumption point of view, and also, they are dimensional rather than categorical instruments; unlike structured interviews, they allow measuring of psychological discomfort in a severity continuum. Moreover, they offer some advantages over clinician-rated scales, as they may take less time, do not require trained personnel, and their administration and scoring process appear to be more standardized. Self-rating scales also require that individuals are able to read at a minimal reading level, and that they speak the language used in at least one translation of the scale. However, some questionnaires also have a cutoff (a threshold) beyond which it is acceptable to assume the presence of a probable depressive disorder. In such cases, a structured interview can be used secondly to test the hypothesis and possibly make a safer and stronger diagnostic hypothesis.

Self-administered tools were also used in most studies investigating the role played by psychosocial factors towards the risk of developing a heart disease, with particular attention to the role played by depression. These were highly standardised and are mostly recommended for an extensive evaluation of cardiac patients [48]. In particular, with regard to depression, they most commonly used were the Hospital Anxiety and Depression Scale (HADS) [26], the Beck Depression Inventory (BDI) [31], and more recently, the nine-items Patient Health Questionnaire (PHQ-9) and the two-items Patient Health Questionnaire (PHQ-2) already described earlier [41–44]. The first three have also been translated and validated in Italian, while the third, although it is highly praised for its excellent psychometric and administration characteristics, it has not been validated neither translated yet into Italian. For more information on the Patient Health Questionnaires it is useful to refer to the relevant literature, as for example, Lichtman et al. [40] or Pozuelo et al. [53].

Finally, although it is the weaker tool from a psychometric point of view, the clinical interview, even if it would only consists of a single question, could also be recommended in the clinical practice as it is easy to use during the initial phases of the cardiac interview. Hence, the interview could simply ask the following questions: “Do you feel down, depressed or discouraged? Did you lose interest or pleasure doing things in everyday life?.” A positive answer at just one of these questions could be indicative of a potential problem which needs further evaluation, such as subministration of a self-administered questionnaire and/or a referral to a psychologist, a psychotherapist or to a psychiatrist, for a specific psychodiagnostic interview.

In general, according to recent Italian, European, and American recommendations [32, 39, 40, 48], in the clinical cardiology practice it would be fruitful to adopt a two-stage approach for the assessment of depression, as well as of any other psychosocial risk factors. Firstly, cardiologists should include some questions on the psychological and social state of the patient such as those descripted above or subminister a self-administered questionnaire in their initial interviews. Subsequently, if in the initial phase some problems have emerge, patients should be referred to qualified personnel (psychologists, clinical psychologists, psychotherapists, and psychiatrists) in order to follow a deeper psychodiagnostic evaluation. Once determined that a patient shows symptoms of emotional or psychosocial distress (like depression), he or she should be proposed to start a specific therapy for that.

Among the tools analysed throughout this paper, that is to say, the Hospital Anxiety and Depression Scale (HADS), the Cognitive Behavioural Assessment Hospital Form (CBA-H), the Beck Depression Inventory (BDI), the two and nine-item Patient Health Questionnaire (PHQ-2, PHQ-9), the Depression Interview and Structured Hamilton (DISH), the Hamilton Rating Scale for Depression (HAM-D/HRSD), and the Composite International Diagnostic Interview (CIDI), two of them appear to be more advantageous within the cardiac unit context. These are the Beck Depression Inventory-II (BDI-II) and the two and nine-items Patient Health Questionnaires, as shown in the summary provided in Table 1, enclosed below.

Though the instruments described earlier may appear to be homogeneous in their strengths and weaknesses, the Beck Depression Inventory-II (BDI-II) and the Patient Health Questionnaires are more efficient towards an early depression assessment within cardiac hospitalised patients for several reasons. Firstly, these instruments are appreciable given their short time completion and for not necessitating the strict presence of trained personnel required. The Beck Depression Inventory-II (BDI-II) and the Patient Health Questionnaires are designed to measure depressive symptoms severity at the present time, hence, embracing health-related quality of life and hospitalisation effects on patients. They are supported by a vast number of studies, becoming well-known and widely used gold-standard tools as they are able to well recognize major depression states and subthreshold depressive disorders too, also closely referring to the DSM-IV manual depression criteria, hence targeting agitation, worthlessness, concentration difficulty and significant energy-loss. Both questionnaires seem to adequately and accurately detect specific conditions that are depression-associated, without failing to conciliate with recent medical and psychological standards like other tools, like the Hospital Anxiety and Depression Scale (HADS) or the Hamilton Rating Scale for Depression (HAM-D/HRSD), do.

Moreover, compared to other tools described earlier like the Hospital Anxiety and Depression Scale (HADS), the Composite International Diagnostic Interview (CIDI), or the Cognitive Behavioural Assessment Hospital Form (CBA-H), they possess strong and clear clinical cutoffs though being rather simple and rapid to be administered and completed. The Beck Depression Inventory-II (BDI-II) and the Patient Health Questionnaires cover symptoms of both atypical and melancholic depression, while atypical symptoms are far less relevant in other instruments such as the Hospital Anxiety and Depression Scale (HADS) and the Composite International Diagnostic Interview (CIDI). Also, the Beck Depression Inventory-II (BDI-II) and the Patient Health Questionnaires assess depression with no contents which are restricted to variables and items possibly confounding by medical illness as the Hamilton Rating Scale for Depression (HAM-D/HRSD) or the Depression Interview and Structured Hamilton (DISH). All in all, most depression measures developed for medically ill populations like cardiac patients have not been adequately tested, while others may present some weaknesses. Amongst the ones selected and described by this paper, the Beck Depression Inventory-II (BDI-II) and the Patient Health Questionnaires appear to be useful and straightforward in evaluating depressive symptoms in terms of presence and severity, with the advantages regarding brevity, format for response options, and good responsiveness to change.

## 3. Limitations of the Review 

This review presents some relevant limitations as the selection of the eight tools proposed entirely refers to specific practice guidelines such as the Italian National System of guidelines (SNLG), the Italian Institute of Health (2005), the American health institutes (NHI), the National Heart, Lung and Blood Institute (2006), and the European guidelines for the prevention of cardiovascular disease in the clinical practice published by the European Cardiology Society (2007). Therefore, other important instruments which are often used in the clinical practice to evaluate depression in patients with cardiovascular disease may have been left out. For example, the paper does not take into account two well-established instruments such as the Primary Care Evaluation of Mental Disorders (PRIME-MD) by Spitzer and colleagues [54] and the General Health Questionnaire (GHQ-12) by Goldberg et al. [55].

These instruments are often used within the primary care setting in order to identify specific mental disorders, though the first fails to adequately classify subthreshold disorders [56], and the second may only be used as screening tools for general dysphoria and social dysfunction as it does not seem to tap into the severity of mental disturbances [57]. Moreover, other measures the present study did not take into account which represent sound assessment tools for depression such as the Centre for Epidemiological Studies Depression Scale (CES-D, [58]) and the Four-Dimensional Symptom Questionnaire (4DSQ, [59]) were not analysed. These are commonly employed tools at the international level which are able to detect depression and depressive disorders in primary care patients. All in all, it is important to point out that further research in the field of mood depression investigation in hospitalised heart disease patients should also consider the instruments previously mentioned in order to fully address other suitable measurements and provide more useful suggestions for health professionals.

## 4. Conclusion

Cardiac patients often display depressive symptoms of some sort following an acute heart event or a cardiac surgery. Also, mood disorders in heart-disease hospitalised individuals represent a high risk factor which may result into premature death. This is why it is particularly important to understand what tools should be used by heart units professionals to efficiently and rapidly detect all forms of possible depression in cardiac patients. There are many different instruments used to measure depression within the cardiac field, of which the vast majority has been recently created or revised. According to the main Italian and international guidelines on mood disorders diagnosis in cardiac patients there are eight principal instruments to be used: the Hospital Anxiety and Depression Scale (HADS), the Cognitive Behavioural Assessment Hospital Form (CBA-H), the Beck Depression Inventory (BDI), the two and nine-item Patient Health Questionnaire (PHQ-2, PHQ-9), the Depression Interview and Structured Hamilton (DISH), the Hamilton Rating Scale for Depression (HAM-D/HRSD), and the Composite International Diagnostic Interview (CIDI). Among these questionnaires, semi-structured or structured clinical interviews, the Beck Depression Inventory-II (BDI-II) and the Patient Health Questionnaires in the two and nine-item version seem to assess any type of mood impairments rapidly and reliably, minimising possible underestimates or misjudgments of the depressive symptomatology from both patients and cardiac units professionals. They are widely used and are supported by past and current literature and represent the gold-standard instruments in the hospitalised setting.

## Figures and Tables

**Table 1:** Depression measures characteristics.

Tool name	Validation study	Number of items	Tool characteristics	Advantages	Disadvantages
Hospital Anxiety and Depression Scale (HADS)	Zigmond and Snaith [26]. The Hospital Anxiety and Depression Scale. Acta Psychiatr Scand, 67(6), 361–370	14 items	Item distribution is perfectly even: 7 items score for depression and 7 for anxiety. The total scale score may be used as a measure of global mood disorder according to the classification of mild (8–10), moderate (11–15), and severe anxiety or depression (16–21) scores. Clinical-practice specific.	Good screening and evaluation of psychological distress symptoms in post-MI patients Sound psychometric properties in MI patients [29] over different time frames (1 or 6 week and 6 months) allowing to determine subscaling of dimensions assessing anhedonia, psychic anxiety, and psychomotor agitation Designed to avoid excessive reliance on common somatic symptoms of illness, fatigue, insomnia, or hypersomnia intertwined with both anxiety and depression but yet different Good performance in screening for separate dimensions of anxiety and depression in cases of anxiety disorders and depression in patients from nonpsychiatric hospital clinics It has at least as good screening properties as similar as but more comprehensive instruments used for identification of anxiety disorders and depression Simple and short Likert-scale scoring	Arbitrary symptom detection due to the constriction of breadth of content, which interferes with providing an efficient first stage screening The depression subscale is weighted towards emotional aspects such as anhedonia rather than sadness; hence, it does not include physical nor cognitive symptoms or suicidal ideation Weak in detecting mood disorders in contexts where many medically ill patients with no psychopathologies, including cardiac units Its use as a depression severity measure is controversial, as cutoffs rely on a tight range Just partially useful in determining whether symptomatology meets formal diagnostic criteria for an anxiety or depressive disorder Its idiosyncratic conception of the core symptom of depression as being anhedonia leads to oversampling and lesser applicability to the mild to moderate range of sad or blue depression symptoms

Cognitive Behavioral Assessment Hospital Forms (CBA-H)	Bertolotti et al. [33]. Il CBA Forma Hospital. In E. Sanavio (Ed.), Le Scale CBA (pp. 158–234). Milano: Cortina	152 items	Card A investigates the emotional reactions at the exact same time of that of the test completion (i.e., hospitalization). It has three scales: state anxiety (A1), health fears (A2), and depressive reactions (A3) Card B includes psychophysical states and sensations perceived by the patient during the previous three months. Card C investigates the psychological variables related to patients' personal traits Card D analyses stable everyday-life behaviour (family/work relationships, general lifestyle)	Cards take into account different time lags, discriminating between emotional states and behavioural changes related to the recent hospitalization or health diagnosis and the patient's preexisting characteristics Final score includes both quantitative and in depth measures as well as suggestions for further interventions as shown patient's global psychological profile which allows us to implement individual hypothesis for eventual additional clinical interview ** **Items in simple dichotomous form, organized into four tabs with simple answering system given by the true/false options Hospital or health context-specific	The CBA-H, developed to allow a quicker assessment within the hospital or health context, has an overall long time completion It is actually a battery of different tests which do not specifically address mood disorders and depressive symptomatology Only Card A is specifically structured to analyse patients' situational psychological state, such as those emotional reactions that the hospitalised individual experiences at the time of completion of the tests Card A is therefore the mainly suitable section for patients who accesses a rehabilitation cardiac program as it enquires about feeling sheltered and being ill, though it may not be enough for clinicians to use the whole CBA-H or to entirely rely on it when assessing a target condition possibly accompanying cardiac patents such as depression

Beck Depression Inventory (BDI-II)	Beck et al. [35]. Manual for the Beck Depression Inventory, 2nd ed. San Antonio, TX	21 items	It assesses the severity of 21 depression symptoms rated on a 4-point scale (0–3). 13 items address cognitive or affective symptoms (hopelessness and guilt). Two of them assess the cardinal symptoms of depression: depressed mood and loss of interest or pleasure in usual activities. The remaining 8 items assess somatic symptoms (insomnia, fatigue, and poor appetite). In screening uses, a total score of 10 or higher is the most widely used cutoff for clinically significant depression. BDI total scores of 10–18 are consistent with mild, 19–29 with moderate, and 30 or higher with severe depression Strong test-retest reliability	Designed to measure depressive symptoms severity at the present time (i.e., hospitalisation) It has a short time completion and does not require trained personnel It pertains to the DSM-IV manual depression criteria, namely, agitation, worthlessness, concentration difficulty, and loss of energy It has been supported by a consisted number of studies, and it is known to correspond with over 90% of clinical diagnoses for patients suffering from depression, hence, becoming a gold-standard tool It adequately covers the range of conditions commonly exhibited by those with depression, measuring the severity of the ailment in an accurate manner, while meeting with recent medical and psychological standards It measures depression intensity on a weekly bases, transversely to the types of depression and different diagnostic categories, as the depressive condition is considered as a psychological trait, therefore nonpathological. That is to say that the score can be analysed in a cognitive-affective subscale and a symptomatic-somatic one Indications of a clinical cut-off alarm are very clear	It measures attitudes and cognitions which are fairly stable over time among depressed patients and may therefore underestimate the degree of improvement during acute pharmacological treatments Because of self-reporting, it could imply participants exaggerating answers; heart disease patients may feel more despondent at the time of the test than they would normally Not strictly suitable as a diagnostic tool as such, better used in conjunction with other tests in order to provide a proper analysis of patients' current mental state

Patient Health Questionnaire (PHQ-2 and PHQ-9)	Kroenke et al. [41]. The PHQ-9: validity of a brief depression severity measure. J Gen Intern Med, 16(9), 606–613 Kroenke et al. [42]. The Patient Health Questionnaire-2: validity of a two-item depression screener. Med Care, 41(11), 1284–1292	2 or 9 items	The Patient Health Questionnaire (PHQ) is a self-administered diagnostic instrument for common mental disorders. The PHQ-9 is the depression module, which scores each of the 9 DSM-IV criteria as “0" (not at all) to “3" (nearly every day) The PHQ-2 is a two-item depression screener which uses 2 items from the PHQ that inquire about the frequency of depressed mood and anhedonia over the past 2 weeks, scoring each as 0 (“not at all") to 3 (“nearly every day")	Both questionnaires are useful tools to recognize not only major depression but also subthreshold depressive disorder in all clinical and nonclinical samples The PHQ-2 is a very simple and rapid yes/no screening tool targeting depression directly and potentially excluding nondepressed patients immediately. A more comprehensive clinical evaluation using the PHQ-9 can be administered simply in the case of a positive answer in the PHQ-2 The PHQ-9 is half the length of many other depression measures, and it refers to the nine criteria of the DSM-IV mood disorders diagnosis The PHQ-9 can provide a depression diagnoses and give a precise value to its symptom severity without leaving out important aspects of health-related quality of life and functional status of hospitalised patients Global score can account for a severity score which can be used for treatment selection and monitoring for coronary artery disease patients Most patients are able to complete the PHQs with no assistance in 5 minutes or just over Brevity and completion easiness are coupled with high construct and criterion validity	Followup of heart disease patients who showed mild sigh of depression is advised, while those with high depression scores should have a specialist reviewing the answers in order to gain a clearer picture Remission signs must be viewed in a rules of thumb logic requiring clinical evaluation of the individual heart disease patient

Interview and Structured Hamilton (DISH)	Freedland et al. [37]. The Depression Interview and Structured Hamilton (DISH): rationale, development, characteristics, and clinical validity. Psychosom Med, 64(6), 897–905	47 Items	It is a structured interview designed to diagnose major and minor depression. The 17-item Hamilton Rating Scale for Depression (HAM-D-17) is also embedded within the DISH to assess severity of depression. Nine of the HAM-D items are rated on a 0–2 scale, and eight are rated on a 0–4 scale. HAM-D total scores can range from 0 to 50. Among medical patients, DISH scores between 10 and 23 are consistent with mild depression and scores of 24 or higher with relatively severe depression It has fair sensitivity to change	Designed to diagnose depression in medically ill patients and to assess its severity DISH diagnosis agrees with the major and minor depressive disorders according to the DSM-IV criteria It allows psychiatric comorbidity assessment	Designed to minimize respondent burden without losing thoroughness nor accuracy it fails to do so as on one hand some contents are option-rigid, while others are of personal preference Symptoms terminology is not fixed and may not find accordance between the patient and the interviewer values or meaning Symptom vary according to how long they last in weeks and they are coded separately according to the number of days they have been present for Only assesses the cardinal symptoms of depression (dysphoria and anhedonia) Rigorous training is needed to be able to subminister the interview Much power is left to the interviewer who arbitrarily decides whether the patient's terms intertwine with the DSM-IV criteria Long time completion of 40 minutes or above Many symptoms cannot be so easily disqualified. And may appear ambiguous symptoms like fatigue unless there is some affirmative evidence that they linked directly to depression

Hamilton Rating Scale for Depression (HAM-D or HRSD)	Williams [47]. A structured interview guide for the Hamilton Depression Rating Scale. Arch Gen Psychiatry, 45(8), 742–747	21 items	The hamilton depression rating scale is a 17-item scale that evaluates depressed mood, vegetative and cognitive symptoms of depression, and comorbid anxiety symptoms. It quantifies the severity of depressive symptomatology It provides partial ratings on current DSM-IV symptoms of depression The 17-items are rated on either a 5-point (0–4) or a 3-point (0–2) scale Test-retest reliability for the HAM-D using the Structured Interview Guide is somehow controversial	The average duration of the HAM-D interviews is 12 minutes It can be used as an indicator of symptoms remission after treatment	Its reliability is low due to use by lay interviewers; the final score is greatly influenced by appropriately trained interviewees It focuses on depression gravity in patients who have already been diagnosed It uses different rating attributed to different symptom domains (like insomnia coded up to 6 points, while fatigue only up to 2) It relies too much on changes which may be related to physiological improvements underestimating emotional and cognitive aspects of depression and overestimating the role of pharmacotherapy It does not have a full overlap with DSM-IV criteria of depression as it does not include exceptions of hypersomnia, increased appetite, and concentration/indecision Noninclusion of all symptom domains related to major depressive disorder, reverse neurovegetative symptoms in particular Some items measure constructs related to irritability and anxiety, loss of interest, and hopelessness which differ from ‘‘pure” depression and may be misleading

Composite International Diagnostic Interview (CIDI)	Wittchen [49]. Reliability and validity studies of the WHO-Composite International Diagnostic Interview (CIDI): a critical review. J Psychiatr Res, 28(1), 57–84	276 Items	A comprehensive and fully standardized diagnostic interview designed for assessing mental disorders with 276 symptom questions, many of which are coupled with probe questions to evaluate symptom severity, as well as questions for assessing help-seeking behavior, psychosocial impairments, and other episode-related questions	It pertains to the syndromic definitions of mood disorders proposed by both ICD-10 and DSM IV Designed to be used by trained interviewers who are not clinicians and it is therefore advantageous in subministration flexibility Its diagnostic sections cover many areas and may be administered independently It performs well as a research instrument to diagnose major depression in MI patients	Primarily intended for use in epidemiological studies of mental disorders hence not referring to hospitalised patients in particular Inflexible and insensitive to change, as it emphasizes lifetime rather than current psychopathology May underdiagnose depressive disorders compared to other instruments. It forces the respondent into a fixed-choice interview format and it may not prove to be a sound instrument within different cultural nor environmental contexts
